# Effect of Precursor Nature and Sol-Gel Synthesis Conditions on TiO_2_ Aerogel’s Structure

**DOI:** 10.3390/molecules26165090

**Published:** 2021-08-22

**Authors:** Jolanta Doneliene, Egle Fataraite-Urboniene, Matas Rudzikas, Saulius Pakalka, Nina Danchova, Juras Ulbikas

**Affiliations:** 1 Applied Research Institute for Prospective Technologies, Vismaliuku st. 34, LT-10243 Vilnius, Lithuania; egle.fataraite@ktu.lt (E.F.-U.); matas.rudzikas@protechnology.lt (M.R.); s.pakalka@met.lt (S.P.); ulbikas@protechnology.lt (J.U.); 2 JSC Modern E-Technologies, Vismaliuku st. 34, LT-10243 Vilnius, Lithuania; 3 Faculty of Mechanical Engineering and Design, Kaunas University of Technology, Studentu st. 56, LT-51424 Kaunas, Lithuania; 4 Department of Physical Chemistry, Faculty of Chemistry and Pharmacy, Sofia University “St. Kliment Ohridski”, J. Bourchier Blvd. 1, 1164 Sofia, Bulgaria; fhndd@chem.uni-sofia.bg

**Keywords:** TiO_2_ aerogel, sol-gel synthesis, solvent exchange, ageing, subcritical drying

## Abstract

The aim of this investigation was to synthesize high porosity TiO_2_ aerogel by applying sol-gel and subcritical drying methods and to identify the influence of reagent’s nature and synthesis conditions on their structural and optical properties. Methods of XRD, FT-IR, BET, STA, SEM, and UV-vis were applied to investigate and compare the properties of synthesized TiO_2_ aerogels and to determine the most effective synthesis route. The structural parameters of the synthesized materials can be varied by changing the precursor type (titanium (IV), isopropoxide (TIP), or tetrabutylorthotitanate (TBOT)) and the nature of the solvent used for additional exchange (*n*-hexane (nH), cyclohexane (CH), or diethyl ether (DE)). All of the subcritical dried samples show the amorphous structure, which tends to crystallize into the anatase phase after calcination. The number of micro and mesopores and the specific surface area depends on the synthesis conditions. The pores with the highest diameter have been found for additionally nH exchanged and aged aerogel synthesized from precursor TIP. Despite the imperfections in the structure, the produced aerogels show structural and optical properties typical of the TiO_2_ structures mentioned in the literature.

## 1. Introduction

Recently, aerogels, as a group of nanomaterials with an immense number of possible applications, have been gaining significant interest in the research community. These compound materials are dried gels with several specific properties such as high specific surface area, high porosity, low apparent density, large volume of open micro and mesopores, high thermal and acoustic insulation, low refractive, and low dielectric constant [1,2,3,4]. Aerogels can be obtained from carbon [5,6,7,8,9], polymers [10,11,12,13,14], or inorganic compounds, namely, SiO_2_, Al_2_O_3_, TiO_2_, ZrO_2_, and others [1,4,15,16,17].

The TiO_2_ aerogel can be obtained by the sol-gel method using special drying methods such as supercritical [18,19,20], sublimation [21], and subcritical [22] drying or drying under ambient conditions [23] to preserve the formed mesopores. The most commonly used method for aerogel drying is supercritical drying, and it requires specific equipment and results in high production costs. As an alternative, subcritical drying is a technically simple and economically viable method for the bulk production of aerogels. It must be noted that TiO_2_ gel has a weaker network skeleton than SiO_2_ gel. Therefore, surface modification [24], gel ageing [22], and additional solvent exchange [25] can be used to prevent the shrinking and cracking of the TiO_2_ gel network and to obtain TiO_2_ aerogel during the drying.

It is a well-known fact that TiO_2_ has three crystallographic phases in nature: brookite, anatase, and rutile. Anatase TiO_2_ shows the best photocatalytic activity because of its valence band characteristics, conduction, and crystal structure [26]. Wide interest in TiO_2_ as a semiconductor [27] can be explained by its promising performance as a heterogeneous photocatalyst for energy and environmental applications, including the photodegradation of organic pollutants for air and water purification [28], the photo-assisted removal of toxic heavy metals [29], the production of solar fuels [30], and the development of self-cleaning surfaces and reflectors [31]. Recently, TiO_2_ aerogels have also been investigated as an electron transport layer in perovskite-based solar cells [20]. Mesoporous titania is widely used as photoanode material in dye-sensitized solar cells (DSSC) because the mesopores (2–50 nm) are capable of encapsulating bulky dye molecules and the permeation of electrolytes that cannot be accomplished using micropores (< 2 nm) [32]. To increase the efficiency of the solar perovskite-based solar module, it would be desirable to maximize the working effective perovskite area by using a mesoporous TiO_2_ aerogel with a high surface area.

This investigation aimed to synthesize high porosity TiO_2_ aerogel by applying sol-gel and subcritical drying methods and to determine the influence of reagent’s nature and synthesis conditions on their structural and optical properties to further their application for the mesoporous network charge carrier material of perovskites during solar cell manufacturing.

The clear understanding of the relation between the precursor type, gel ageing, additional solvent exchange, and other synthesis parameters that control the formation mechanism of TiO_2_ aerogel and affect its properties remains an unsolved issue. Sufficiently good results are obtained for TiO_2_ aerogels after supercritical drying [18,19,22], while the results after subcritical drying, as a more cost-effective technology, are quite limited, and the properties of synthesized products are lower compared with the supercritical dried ones. In this study, the structural properties of subcritical dried (400 mbar, 70 °C, 8 h) TiO_2_ aerogels were obtained by using two types of precursors (titanium (IV) isopropoxide (TIP) or tetrabutylorthotitanate (TBOT)). The obtained gel network was very flexible. The effect of ageing (72 h, 40 °C) on gel stiffness has been evaluated. The additional exchange in low surface tension solvent (n-hexane (nH), cyclohexane (CH), or diethyl ether (DE)) was applied to determine a more stable network structure. The properties of the synthesis products were evaluated by applying the methods of X-ray diffraction (XRD), Fourier transform infrared spectroscopy (FT-IR), the Brunauer, Emmet, and Teller (BET) method, simultaneous thermal analysis (STA), scanning electron microscopy (SEM), and ultraviolet-visible (UV-vis) spectroscopy, which are discussed in Section 2—Results and Discussions. A more detailed materials characterization and test methodology are presented in Section 3—Materials and Methods. The conclusions are presented separately in Section 4.

## 2. Results and Discussions

### 2.1. XRD Analysis

XRD analysis was carried out to evaluate the structural changes of synthesized TiO_2_ aerogels in dependence of precursor nature (TIP and TBOT), ageing (72 h, 40 °C), the solvent type used for additional solvent exchange (nH, CH, DE), and subcritical drying (400 mbar, 70 °C, 8 h). As expected, probably due to the low temperatures of sol-gel synthesis and subcritical drying conditions, all of the investigated TiO_2_ aerogel samples show the amorphous structure, and a more detailed structural characterization by this method cannot be implemented (Supplementary Materials; Figure S1). [33,34]. For this reason, other analysis methods (STA, FT-IR, BET, SEM, granulometric analysis, and UV-vis) were applied for the synthesis products characterization.

### 2.2. Thermal Analysis

**Figure 1:**
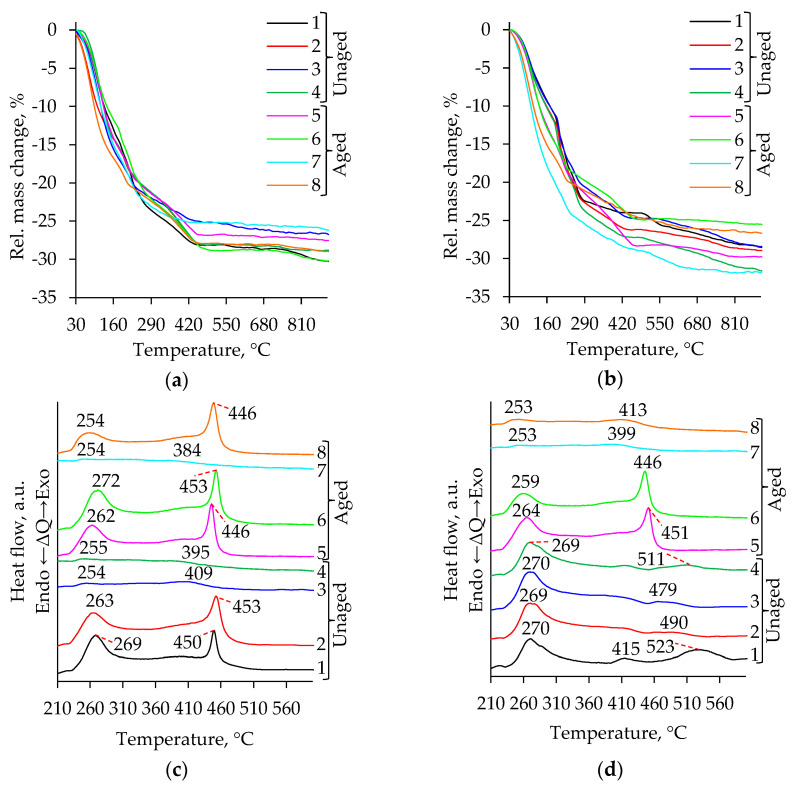
TG (**a**,**b**) and DTA (**c**,**d**) curves of TiO_2_ aerogels after subcritical drying in dependence of precursor type (TIP (**a**), TBOT (**b**)), ageing (without aging (**1**–**4**), aged (**5**–**8**)) and solvent used for additional solvent exchange (**1**, **5**—without exchange; **2**, **6**—CH; **3**, **7**—nH; **4**, **8**—DE).

Figure 1 a and b show the thermogravimetric (TG), and c and d show the differential thermal analysis (DTA) curves of the synthesized titania aerogels. The TG curves show a mass loss below ~ 225 °C. This can be attributed to the adsorbed impurities and moisture, while a mass loss above 225 °C can be related to the decomposition of the organic groups (–CH_2_ and –CH_3_) [33] and to the fracturing of the –OH groups [24].

The obtained total mass loss was found to vary between 27–30% and 26–33% for samples prepared using precursors TIP (Figure 1a) and TBOT (Figure 1b), respectively. This means that during subcritical drying, the decomposition of organic groups and solvent residuals were not fully reached. This situation differs from supercritical drying conditions, where higher temperatures were applied, the decomposition of organic groups was obtained during synthesis, and the mass loss in TG curves reached approximately up to 5% [33].

According to the weight-loss trend in the TiO_2_ aerogel (Figure 1a,b; Table S1), the decrease that started at about 228 °C and continued to ~350 °C and the exothermic peak in the same temperature range (Figure 1c,d; Table S1) corresponds to the removal of solvents and alkoxy groups [35,36]. The STA analysis shows that the exothermic peaks in the temperature range of 366–560 °C were formed (Figure 1c,d; Table 1). Such kinds of peaks are characteristic of anatase formation and were observed in all samples [22]. These peaks can be attributed to the conversion of Ti(OH)_4_ to TiO_2_ (dehydroxylation) and occurred during the crystallization of anatase [35,36].

**Figure 2:**
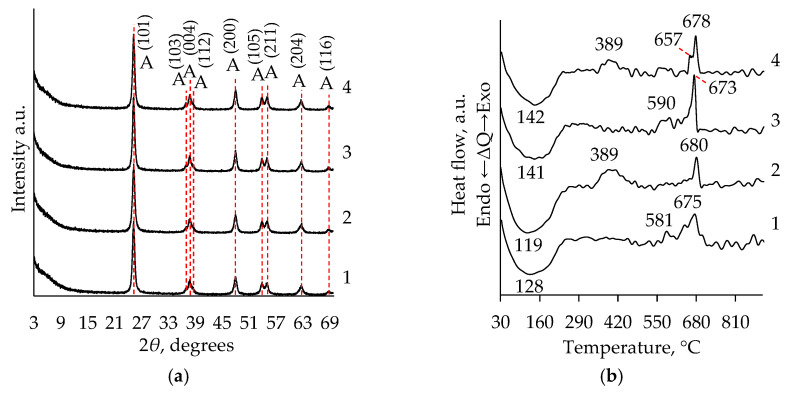
Effect of calcination on XRD (**a**) and DTA (**b**) patterns of TiO_2_ aerogel ((**1, 3**) without ageing; (**2, 4**) after ageing. **1**, **2** curves correspond to precursor TIP and **3**, **4**—to TBOT. Indexes: A—anatase phase.

To confirm the contribution of the determined exothermal peaks in the range of 366–560 °C in the DTA curves to the anatase phase formation, part of the samples was calcined at 500 °C for two hours. The temperature of the thermal treatment was selected according to the DTA results, presented in Figure 1 and Table 1, and according to the information found in the literature that the anatase phase formation takes place at temperatures higher than 400 °C [21,22,33]. The calcination temperature was selected, ensuring possible thermal conversions in the mentioned temperature range. XRD analysis was carried out for calcinated samples. Obtained XRD patterns (Figure 2a) of calcinated TiO_2_ aerogels samples were matched to the reference patterns for the corresponding oxide (PDF-00-064-0863, not shown), and presumption about the crystallization into the anatase phase have been confirmed [22]. The relative intensity of peaks in the XRD pattern (Figure 2a) of the calcinated samples remains the same in all investigated cases.

To evaluate the organic groups decomposition, calcinated samples were also investigated by STA analysis (Figure 2b). The endothermic peaks visible in all samples at the temperature range of 119–142 °C can be related to the moisture removal. In the temperature region of 225–300 °C, the creation of peaks was not observed, indicating that most organic group decomposition is finalized during the calcination process. In the DTA curves (Figure 2b, curves 2 and 4) for the calcinated aged samples at 389 °C temperature, the visible exothermic peak can also be related to the formation of anatase. This means that after the calcination of the aged samples, the amorphous phase transformation to crystalline anatase was not completed. These peaks in the curves for the samples without ageing ((Figure 2b, curves 1 and 3) were not found. The visible exothermic peaks in the temperature range of 540–695 °C can be attributed to the conversion of anatase to rutile (Figure 2b) [25,37].

### 2.3. FT-IR Analysis

**Figure 3:**
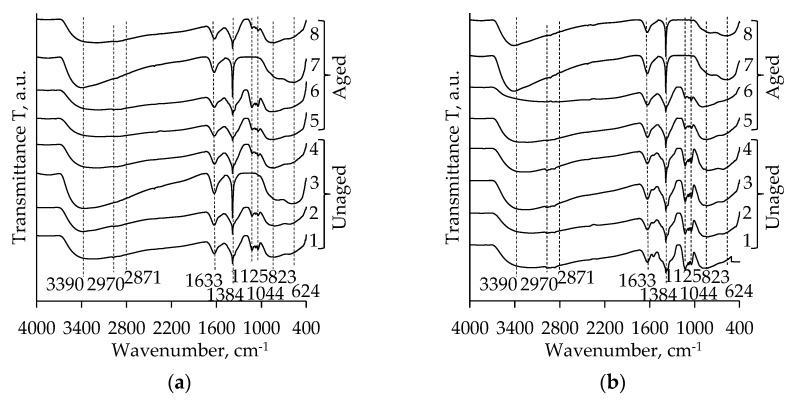
FT-IR spectra of TiO_2_ aerogels after subcritical drying without aging (**1**–**4**) and after 72 h aging (**5**–**8**) in dependence of precursor type (TIP (**a**), TBOT (**b**)) and solvent used for additional solvent exchange (**1**, **5**—without exchange; **2**, **6**—CH; **3**, **7**—nH; **4**, **8**—DE).

Figure 3 shows the FT-IR spectra of unaged and aged TiO_2_ aerogels synthesized using the precursors TIP and TBOT and additional solvent exchange (nH, CH or DE). There are no significant differences between the curves. The broad absorption bands at ~ 3390 cm^−1^ and ~ 1633 cm^−1^ are attributed to the stretching vibrations of the hydroxyl (OH) groups on the surface and the bending vibrations of the adsorbed water molecules (H–O–H), respectively [38,39,40,41]. The peaks at 2970 and 2871 cm^−1^ of the TiO_2_ aerogels belong to the CH_2_ symmetric stretching and the CH_3_ symmetric stretching of the remaining organic compounds (like dissociative n-hexane (nH) and EtOH) in the pores of the TiO_2_ aerogels, respectively [38,40,42]. The absorption peak at 1380 cm^−1^ can be attributed to the CH_3_ symmetric deformation vibration [42]. Weak peaks at 1040–1120 cm^−1^ are due to the stretching vibration of C–C bonds [40,42].

The broad bands between 400 and 1000 cm^−1^ in the TiO_2_ aerogel spectrum are related to the bending vibration of Ti–O–Ti and the stretching vibration of Ti–O [43,44,45]. However, the broad peaks in the mentioned range could also be attributed to a combination of the Ti–O–Ti and Ti–O–C bond stretching vibrations [46]. It should be noted that the absorption peaks attributed to TiO_2_ are more intensive for *n*-hexane exchanged aerogels.

**Figure 4:**
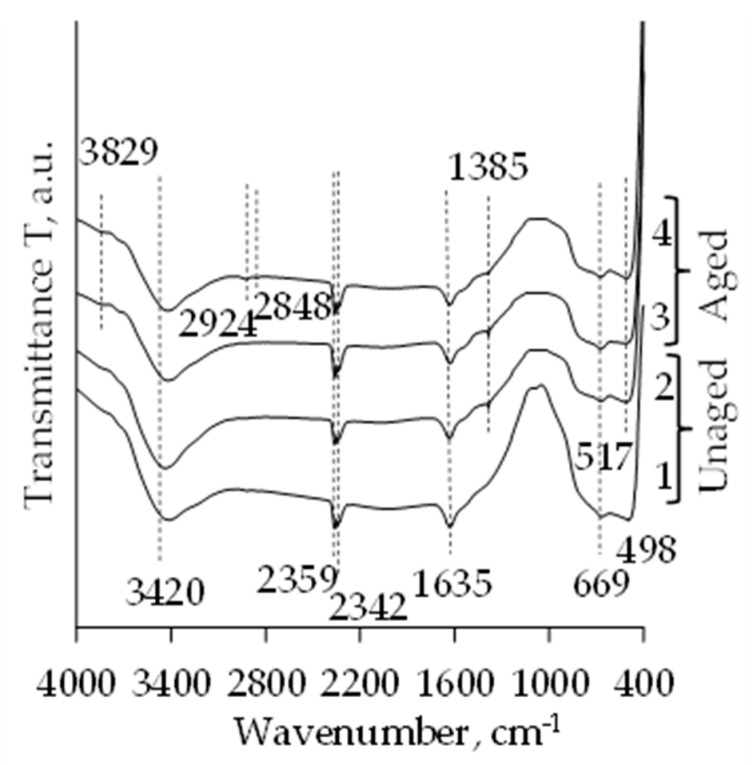
FT-IR spectra of subcritical dried and calcinated TiO_2_ aerogels ((**1, 2**) unaged; (**3, 4**) aged; **1**, **3** curves correspond to precursor TIP and **2**, **4** correspond to precursor TBOT).

The intensity of the absorption bands characteristic of TiO_2_ increases after calcination as well (Figure 4). The bands attributed to Ti–O stretching vibration appear at 669 cm^−1^. The band at 450 cm^−1^ and 517 cm^−1^ corresponds to the superimposing of Ti–O bending vibration. These bands also confirm the crystallization and the transition to the anatase phase. The absorption bands (at 2970, 2871, 1380, 1040–1120 cm^−1^), earlier attributed to the residual organic groups are significantly reduced or entirely disappear after calcination [32,39,41].

### 2.4. BET Analysis

**Figure 5:**
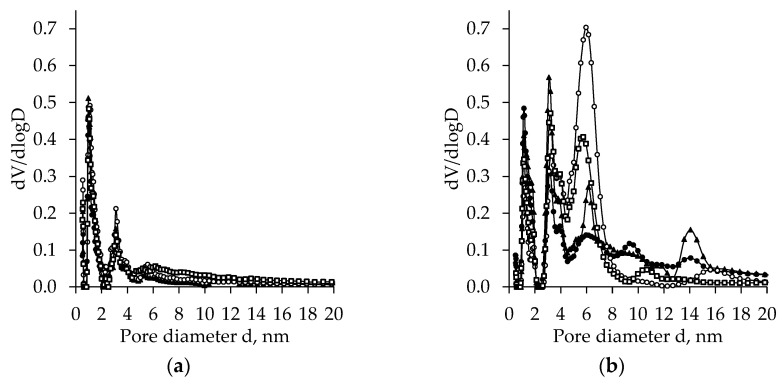
Pore size distribution of the subcritical dried TiO_2_ aerogel synthesized using precursor TBOT vs. exchanging solvent type (● –without exchange, ◯ –nH, ▲ –cH, □ –DE)—without (**a**) and after (**b**) ageing.

**Figure 6:**
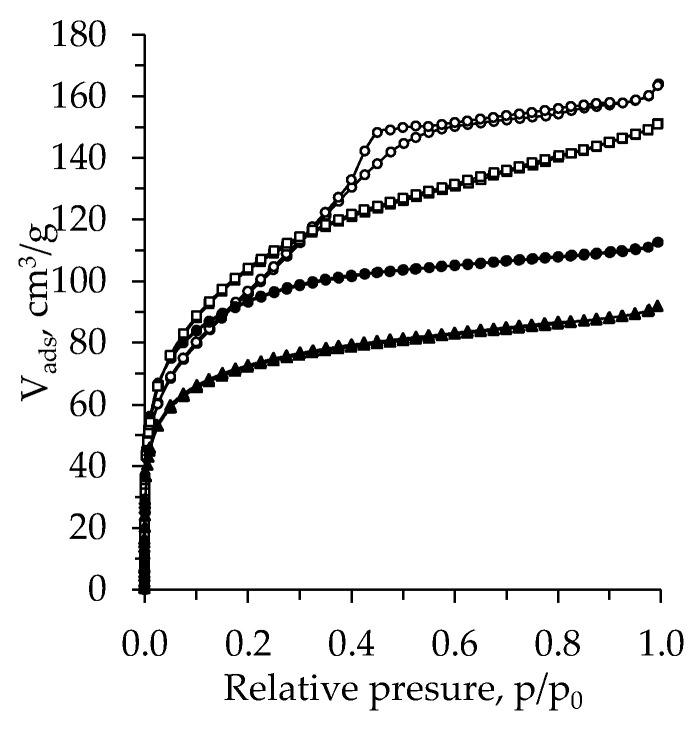
Isotherms for subcritical dried TiO_2_ aerogel synthesized using precursor TBOT and additional nH exchange (unaged (▲ –without exchange, ● –nH) and aged (□ –without exchange, ◯ –nH)).

**Figure 7:**
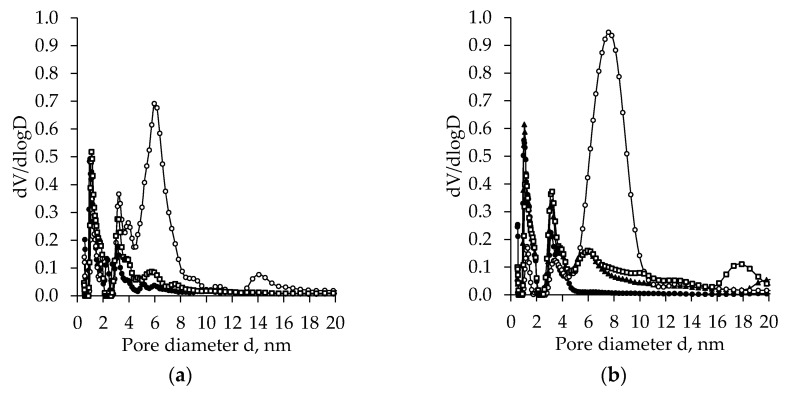
Pore size distribution of subcritical dried unaged (**a**) and aged (**b**) TiO_2_ aerogel synthesized using the precursor TIP and additional solvent exchange (● –without exchange, ◯ –nH, ▲ –cH, □ –DE).

**Figure 8:**
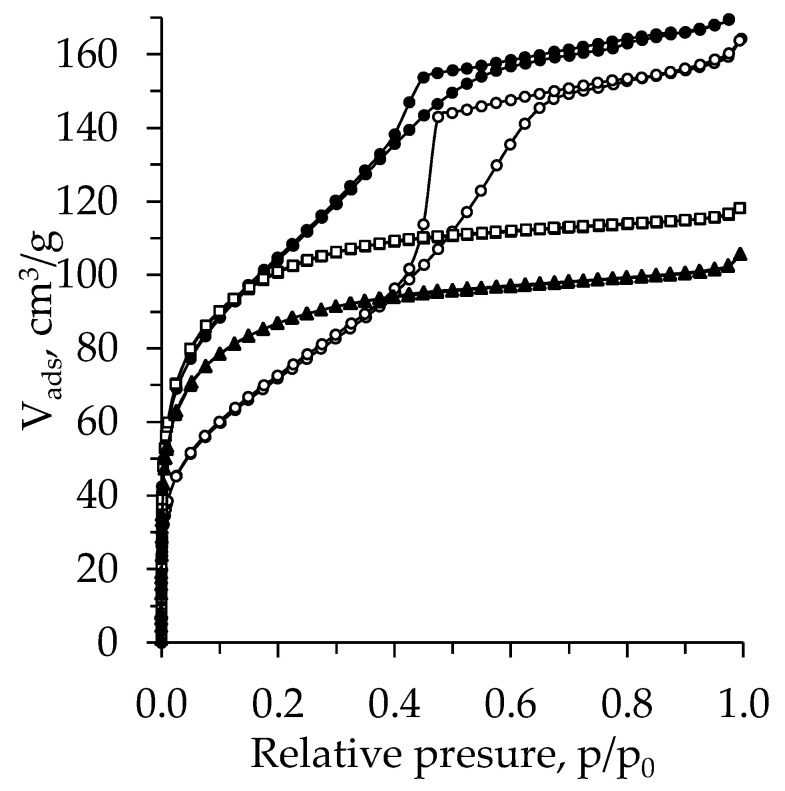
Isotherms for subcritical dried TiO_2_ aerogel synthesized using the precursor TIP and additional nH exchange (unaged (▲ –without exchange, ● –nH) and aged (□ –without exchange, ◯ –nH)).

**Figure 9:**
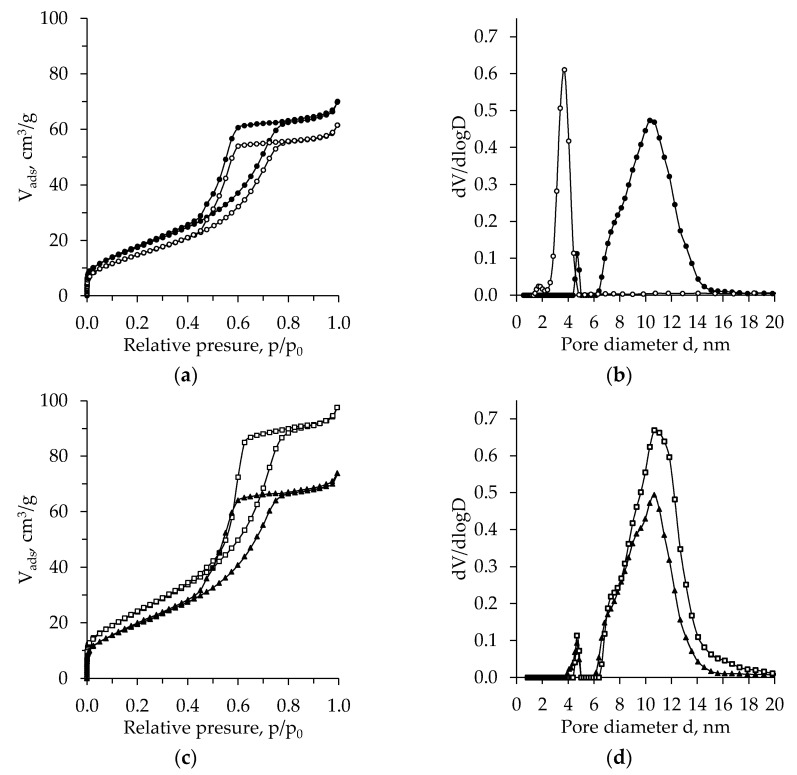
The BET adsorption–desorption isotherms (**a**,**c**) and pore size distribution (**b**,**d**) of calcinated TiO_2_ aerogel vs. precursor (TBOT (**a**,**b**), TIP (**c**,**d**) and aging (●, ▲ –unaged, ◯, □ –aged).

Figure 5, Figure 6, Figure 7, Figure 8 and Figure 9 present the BET results for subcritical dried unaged and aged TiO_2_ aerogels synthesized using the precursors TBOT and TIP and the additional solvent exchange (CH, nH, or DE).

Subcritical dried unaged TiO_2_ aerogels synthesized from TBOT indicate a relatively narrow bi-modal pores size distribution in the range of 0–5 nm (Figure 5a). The effect of additional solvent exchange is negligible, and all peaks were found at pores with a diameter of 0.97 and 3.10 nm, and the peaks of pores up to 0.97 nm were more than two times higher than those for 3.10 nm. The comparison of curves obtained after ageing with unaged ones indicates that ageing changes the porosity of subcritical dried TiO_2_ aerogels (Figure 5b). After ageing TiO_2_ aerogels synthesized using precursor TBOT, highly expressed polydispersity is indicated, where pore size distribution changes from bimodal to trimodal, with a decrease in the first peak height (at a nanopore diameter of about 1.0 nm) and a significant increase of the second peak at a pore diameter of 3.09 nm and an evident appearance of the third peak at 5.92 nm. The height of the third peak was found to be highly dependent on the solvent type used for exchange. The highest and widest third peak was found for additionally nH exchanged TiO_2_ aerogels. This peak is about 2–6 times higher compared to the TiO_2_ aerogels exchanged in other solvents. Besides, ageing increases the number of pores with diameters in the range of 8–16 nm.

The N_2_ adsorption–desorption isotherms of the TiO_2_ aerogel prepared from TBOT are presented in Figure 6. According to the IUPAC classification [47,48], the obtained isotherms can be classified as a type-I with an H4 hysteresis loop, excluding the aged and nH exchanged sample, for which a type-IV isotherm with an H2 hysteresis loop was found. A type-I isotherm and H4 hysteresis loop indicate that the synthesized structure has a microporous and mesoporous structure with narrow slit pores. These data correlate well with the obtained pore size distribution curves presented in Figure 5. A type IV isotherm and H2 hysteresis found for additionally nH exchanged aerogel are typical for the solids with mesopores and micropores [49], whose structure is disordered and the distribution of pore size and shape is not well defined [47,48,49].

In Figure 7, there are plots of the pore size distribution for the TiO_2_ aerogel synthesized using the precursor TIP and the additional solvent exchange (nH, CH, or DE). As in the previously discussed case, an apparent effect of solvent nature, as well as ageing, was found. Pore diameter distribution curves also are multimodal. The curves for nH exchanged TiO_2_ aerogels indicate the creation of a bigger diameter of pores compared to the other ones. The highest peak in the region of pore diameter 5–10 nm was found for both unaged and aged samples. While the peak for aged samples is higher compared to those for unaged ones, the pore diameter distribution curves also show small peaks in the region of 13–16 nm for unaged samples, which after ageing was shifted to the zone of larger values, i.e., 16–20 nm. The effect of ageing on aerogel pore size distribution is insignificant when additional exchange in other solvents (CH or DE) was used, and it was very close to those obtained for unaged samples.

Figure 8 presents the N_2_ adsorption–desorption isotherms for the TiO_2_ aerogel prepared from TIP. The isotherms for the TiO_2_ aerogels without additional solvent exchange can also be attributed to the type-I isotherms with an H4 hysteresis loop. As in the TBOT case, the nH exchanged TiO_2_ aerogels attained type-IV isotherms with an H2 hysteresis loop [47,48]. The hysteresis loop for the nH exchanged aged aerogel is wider than for the unaged and nH exchanged samples. That coincides well with the pore distribution curves (Figure 7). The curve peaks of pore size distribution are shifted toward a larger pore diameter zone after ageing.

For comparison, BET analysis was also carried out for the calcinated TiO_2_ aerogels without additional solvent exchange. The obtained isotherms and pore size distribution are presented in Figure 9. For both precursor cases, the isotherms type after calcination changes from type-I with an H4 hysteresis loop to type-IV isotherm with an H2 hysteresis loop [47,48]. The hysteresis loops (Figure 9a,c) are significantly wider compared to the samples before calcination (Figure 6 and Figure 8), and after calcination, the adsorption–desorption intersection zone shifts to lower relative pressure p/p_0_ values. Meanwhile, the quantity of adsorbed V_ads_ for the samples obtained with precursor TIP was higher than for TBOT and indicated the creation of the mesoporous structure. The calcination results in a more even pore size distribution, one highly expressed peak, and the evident effect of precursor nature and ageing (Figure 9b,d). For unaged calcinated TiO_2_ aerogel synthesized using TBOT, the pore size distribution curves have a high peak in the region of pores with a diameter of 7–15 nm and only a small narrow peak at 5 nm. For aged TiO_2_ calcinated aerogel, the pore diameter distribution curve shows a very narrow high peak in the region of 2.5–5 nm (Figure 9b). Using the precursor TIP effect of ageing on calcinated aerogel, the pore size distribution is not very significant (Figure 9d). For unaged and aged calcinated aerogels, the pore diameter distribution curves show two peaks of different heights: the lower one was found in the region of 4–5 nm and the higher one in the region of 6–15 nm. There, the highest value of pore diameter was 11 nm. For aged samples, the number of these pores was found to be 1.4 times higher compared to the calcinated unaged samples.

The summary of other TiO_2_ aerogel parameters obtained during the BET test is presented in Table 2 and Table 3. The comparison of them indicates that calcination results in a significant decrease in the specific surface area [1] and in increased pore size. In contrast, the ageing effect on the TiO_2_ aerogel pore size is highly dependent on the precursor and solvent used for additional exchange, and clear dependence is difficult to identify.

**Figure 10:**
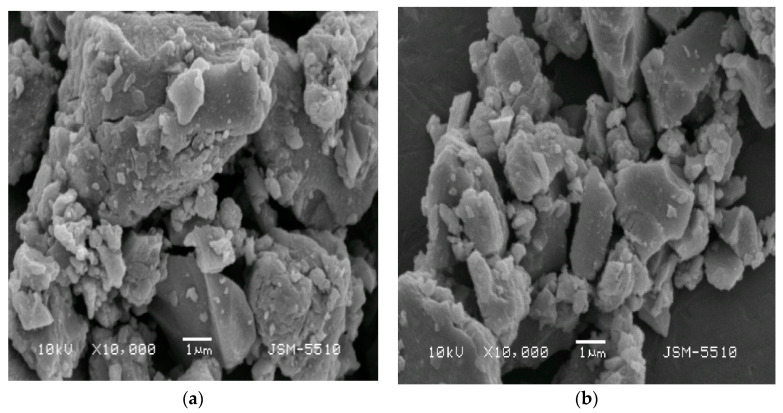
SEM images of calcinated aged TiO_2_ aerogel in dependence of precursor type (TIP (**a**), TBOT (**b**)).

The microstructure of calcinated aged samples is typical for TiO_2_ aerogels (Figure 10) [26,50,51]. The particles are highly polydispersed and tend to agglomerate.

### 2.5. UV-Vis Analysis

**Figure 11:**
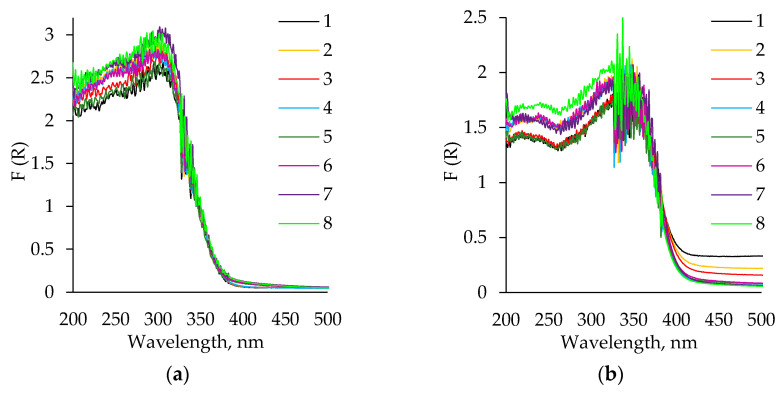
Diffuse reflectance spectra of TiO_2_ aerogel powders: (**a**) before calcination; (**b**) after calcination. 1–4 curves correspond to precursor TBOT and 5–8 to TIP; 1, 3—without aging and solvent exchange; 2, 4—aged, without solvent exchange; 5, 7—unaged, nH exchanged; 6, 8—aged, nH exchanged.

UV-vis analysis was carried out to evaluate the optical properties of the synthesized aerogels. All of the obtained spectra are typical for catalytic titania powders and consist of a strong UV absorption with a shoulder at about 335 nm (Figure 11a).

Some samples after calcination display a light grey coloration due to a broad, weak transition in the visible spectral region. Such grey coloration can be attributed to the surface defects / doping impurities in oxides and is often caused by surface diffusion. The calcination of the TiO_2_ aerogel powders leads to an absorption range shift to a longer wavelength by about 30 nm (Figure 11b) [52,53]. A similar wavelength shift has been reported for zirconia gels modified with organic agents [54].

**Figure 12:**
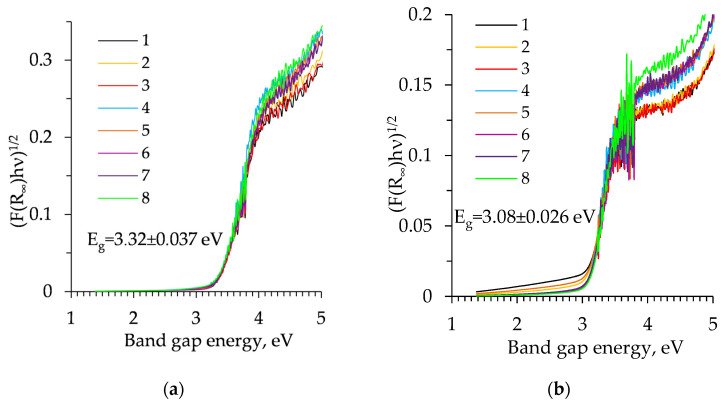
Tauc plot of TiO_2_ aerogel powders: (**a**) before calcination; (**b**) after calcination (**1**–**4** curves correspond to precursor TBOT and **5**–**6** to TIP; **1**, **3**—unaged and without solvent exchange; **2**, **4**—aged and without solvent exchange; **5**, **7**—unaged and nH exchanged; **6**, **8**—aged and nH exchanged).

The optical bandgap energy E_g_ of heat untreated and calcinated samples were calculated by the Tauc Plot method (Figure 12).

It was calculated that the indirect bandgap energy (E_g_) of uncalcined samples was 3.32 eV, with a standard deviation of 0.037 eV. The bandgap energy of amorphous aerogels is slightly higher compared to crystalline anatase [55]. It should be noted that the bandgap energy (E_g_) of the calcinated samples decreased to 3.08 eV (the standard deviation was 0.026 eV). The obtained results are consistent with the literature data that anatase as an indirect bandgap semiconductor has bandgap energy of between 3–3.2 eV [55,56,57,58].

## 3. Materials and Methods

### 3.1. Materials

In this paper, the following reagents were used: titanium (IV) isopropoxide (TIP, 98% Fluorochem, UK), tetrabutylorthotitanate (TBOT, 95% Fluorochem, Hadfield, UK), nitric acid (HNO_3_, 65%, Chempur, Piekary Śląskie, Poland), ethanol (EtOH, 99.5%, Emparta ASC, Darmstadt, Germany), distilled water, *n*-hexane (nH, 99%, Chempur, Poland), cyclohexane (CH, 99%, Chempur, Poland), and diethyl ether (DE, 99.5%, Chempur, Poland). All reagents were used without further purification.

### 3.2. TiO_2_ Aerogels Synthesis

The TiO_2_ gels were prepared with the acid-catalyzed sol–gel method using TIP or TBOT, HNO_3_, EtOH, and H_2_O, with a molar ratio of 1:0.08:21:7.35, respectively. The titania precursor (TIP or TBOT) was dissolved in the EtOH (Solution A). HNO_3_, EtOH, and distilled water were mixed (Solution B). Both of the obtained solutions were stirred intensively for 20 min. Solution B was added to Solution A under intense stirring. The wet gels were formed in 1–2 s. Half of all of the obtained gels were additionally aged at 40 °C for 72 h. The unaged and aged gels were solvents exchanged twice in EtOH at 40 °C for 24 h to remove water from the gel network. Part of obtained alcogels was additionally exchanged in selected solvents: *n*-hexane, diethyl ether, or cyclohexane at 40 °C for 24 h. After the solvent exchange, the samples were washed with acetone. The drying of the gel network was performed at the subcritical condition of the pressure in a vacuum oven (400 mbar) at 70 °C for 8 h (*VC50* (*SalvisLAB*, Rotkreuz, Switzerland) with the volume of 50 L, vacuum system *Vacuubrand PC 8 / RC 6* (*Vacuubrand GMBH + CO KG,* Wertheim, Germany); max. pumping speed was 5.9/6.9 m^3^/h). The synthesis products were sieved through a sieve with a mesh width of 80 µm. Additionally, some of the dried gels were thermally treated at 500 °C for 2 h (*SNOL 10/1300, SnolTherm* business unit, part of *Umega Group, AB*, Utena, Lithuania, with a heating rate of 4 °C/min).

### 3.3. Characterization

Methods of X-ray diffraction (XRD), simultaneous thermal analysis (STA), Fourier transform infrared spectroscopy (FT-IR), Brunauer, Emmet, and Teller (BET), scanning electron microscopy (SEM), and ultraviolet–visible spectroscopy (UV-vis) were applied to investigate and compare the properties of synthesized TiO_2_ aerogels and to determine the most effective synthesis route. XRD analysis was performed by using the *D8 Advance* diffractometer (*Bruker AXS*, Karlsruhe, Germany) operating at the tube voltage of 40 kV and tube current of 40 mA. The X-ray beam was filtered with a Ni 0.02-mm filter to select the CuK_α_ wavelength. The diffraction patterns were recorded in a Bragg–Brentano geometry using a fast counting detector *Bruker LynxEye* based on the silicon strip technology. The specimens were scanned over the range of 2*θ* = 3–70° at a scanning speed of 6°/min using a coupled two theta/theta scan type.

STA (differential scanning calorimetry—DSC and thermogravimetry—TG) was also employed to measure samples thermal stability and phase transformation at a heating rate of 15 °C/min, when the temperature ranged from 30 °C up to 950 °C under air atmosphere. The test was carried out with the *Linseis instrument STA PT1000*. Ceramic sample handlers and platinum crucibles were used.

The specific surface area was determined by the Brunauer, Emmet, and Teller (BET) method. Measurements were performed on an *Autosorb iQ* (*Quantachrome Instruments*, Boynton Beach, Fla., USA) using an N_2_ gas adsorption isotherm at 77 K.

SEM investigations were performed using a standard electron microscope *JEOL 5510* working on SE regime. Particles were Au-covered.

FT-IR analysis was performed on a *Perkin Elmer FT-IR System* spectrometer (*Perkin Elmer*, USA) in the main infrared spectrum range of 4000–400 cm^−1^ (±0.01 cm^−1^). The tablet-shaped samples were pressed in a vacuum press from the mixture of 1 mg of the test substance and 200 mg of KBr.

**Figure 13:**
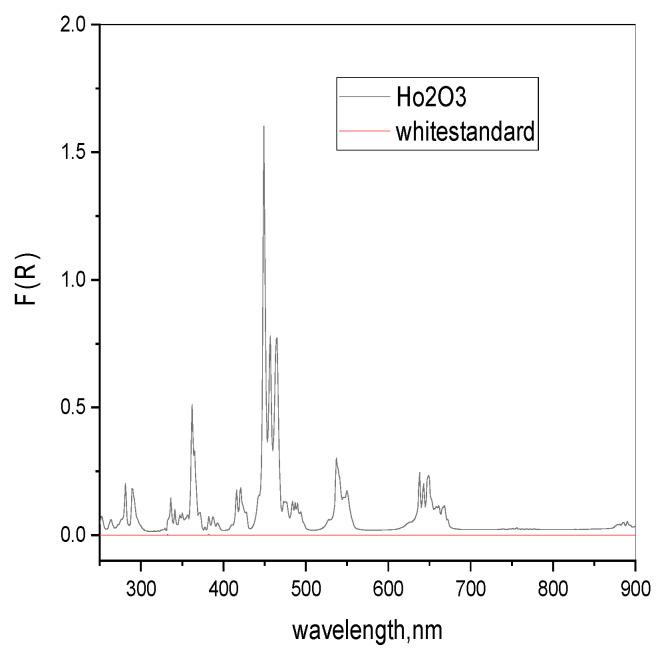
Diffuse reflectance spectra of Ho_2_O_3_ powders, together with the baseline used here / white certified reflectance standard.

Room temperature diffuse reflectance spectra were measured on a *Perkin Elmer* (Walham, MA, USA,) *Lambda 35* spectrophotometer equipped with a reflectance accessory (*RSA-PE-20*, *Labsphere*, North Sutton, NH, USA) and a vertical sample holder with a quartz glass window between 250 nm and 900 nm. As a reference, white and black certified reflectance standards *Labsphere^®^* were used. The f-f transitions of Ho_2_O_3_ micro powders were used as a reference. Peak maxima and intensities were in good agreement with the theory. From the measured diffuse reflectance R (%), the Kubelka-Munk function F(R) has been calculated [59]. The diffuse reflectance spectra of Ho_2_O_3_ powders are visualized in Figure 13.

The Tauc Plot method was used to determine the optical energy bandgap (E_g_) of selected samples. The optical absorption strength depends on the difference between the photon energy and the bandgap as:

(*F(R)hν*)^1/n^ = *A*(*hν* − *E_g_*), (1)

where *h* is Planck’s constant, *ν* is the photon’s frequency, for indirect allowed transitions *n* = 2, *E_g_* is the bandgap, and *A* is the slope of the Tauc plot in the linear region [60].

## 4. Conclusions

The structure of TiO_2_ aerogels synthesized by applying the sol-gel method and subcritical drying conditions have been investigated. The effects of titanium precursor (TIP and TBOT), solvent (nH, CH, DE) used for additional solvent exchange, ageing, and calcination have been evaluated. It was found that all subcritical dried samples show amorphous structures, which tend to crystallize into the anatase phase after calcination. All synthesized aerogels are highly polydispersed systems with a variation of pore size in the region of 0.5–17 nm. The number of micro and mesopores and the specific surface area *S*_BET_ of aerogels are highly dependent on the synthesis conditions. The pores with the highest diameter have been found for additionally nH exchanged, aged aerogel synthesized from precursor TIP. The calcination significantly decreases the specific surface area *S*_BET_ and increases the pore sizes of subcritical dried samples without additional solvent exchange. Despite the imperfections in the structure, the produced TiO_2_ aerogels show structural and optical properties typical of the TiO_2_ structures mentioned in the literature. The obtained results seem promising for the application of synthesized aerogels for photovoltaic purposes. Further investigations are planned to evaluate their suitability in terms of their application in perovskite solar cells.

## Figures and Tables

**Table 1:** The main characteristics of the thermal effects typical of anatase formation.

Precursor	Ageing Duration, h	Solvent	T_onset_, °C	T_max_, °C	T_end_, °C	Heat of Process, J/g	Mass Change, %
TIP	0	-	439.6	449.4	458.8	464.98	2.832
		CH	433.7	452.7	466.2	807.51	5.169
		nH	371.1	409.0	438.3	190.80	1.498
		DE	369.4	395.2	427.8	88.94	1.264
	72	-	433.6	445.5	454.8	567.30	3.187
		CH	439.8	452.8	462.5	702.34	4.752
		nH	371.9	383.6	440.5	64.52	0.663
		DE	430.5	445.6	457.4	665.72	4.055
TBOT	0	-	488.6	523.4	559.9	336.23	1.841
		CH	452.1	490.2	522.1	56.54	0.217
		nH	452.1	479.0	515.3	67.1	0.017
		DE	454.2	511.0	544.7	151.95	1.002
	72	-	434.9	451.1	460.9	596.68	4.021
		CH	430.7	445.8	457.0	631.05	3.661
		nH	366.7	399.3	436.4	156.37	2.039
		DE	371.2	413.6	452.3	260.99	1.883

**Table 2:** Morphological parameters of TiO_2_ aerogels vs. precursor nature, solvent type, and ageing duration.

Precursor	Ageing Duration, h	Solvent	Surface Area, m^2^/g	Pore Volume, cc/g
TIP	0	-	318.597	0.146
		CH	331.262	0.165
		nH	374.564	0.247
		DE	313.811	0.151
	72	-	367.579	0.167
		CH	384.475	0.207
		nH	263.443	0.234
		DE	366.314	0.213
TBOT	0	-	264.054	0.146
		CH	302.115	0.165
		nH	340.861	0.247
		DE	296.781	0.151
	72	-	378.066	0.216
		CH	423.731	0.245
		nH	354.656	0.233
		DE	377.213	0.224

The difference in measured values obtained from repeated runs was found to be lower than 5%.

**Table 3:** Morphological parameters of calcinated aerogels vs. precursor nature and aging duration.

Precursor	Ageing Duration, h	Surface Area, m^2^/g	Pore Volume, cc/g
TIP	0	64.523	0.104
	72	80.582	0.140
TBOT	0	58.774	0.098
	72	47.802	0.087

## Data Availability

Data sharing is not applicable.

## Supplementary Materials

The following are available online. Figure S1: XRD patterns of TiO2 aerogels after subcritical drying without aging (1–4) and after aging (5–8) in dependence of precursor type (TIP (**a**), TBOT (**b**)) and solvent type used for additional solvent exchange (1, 5—without exchange; 2, 6—CH; 3, 7—nH ; 4, 8—DE); Table S1. The main characteristics of thermal effects are attributed to the organic groups decomposition.

## Sample Availability

Samples of the compounds are not available from the authors.
