# Supplementary material for: Effect of Precursor Nature and Sol-Gel Synthesis Conditions on TiO2 Aerogel’s Structure
Source: Molecules. 2021 Aug 22;26(16):5090. doi: 10.3390/molecules26165090 (PMC8401230; doi:10.3390/molecules26165090)
Supplement: Supplementary file 1 [file molecules-26-05090-s001.zip › molecules-1297318-supplementary.pdf]

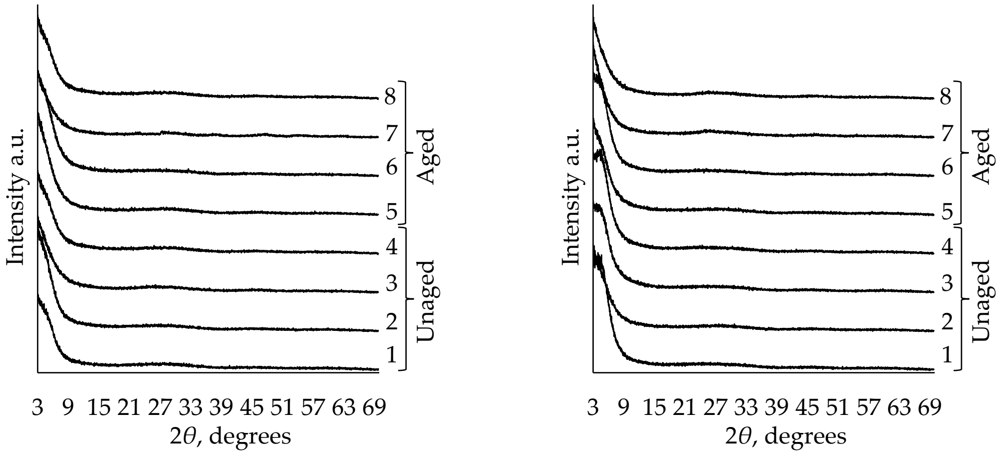

(**a**) (**b**)

Figure S1. XRD patterns of TiO_2 aerogels after subcritical drying without aging (**1-4**) and after aging (**5-8**) in dependence of precursor type (TIP (**a**), TBOT (**b**)) and solvent type used for additional solvent exchange (**1**, **5** – without exchange; **2**, **6** – CH; **3**, **7** – nH ; **4**, **8** – DE).

Table S1. The main characteristics of thermal effects attributed to organic groups decomposition.

Precursor	Aging duration, h	Solvent	T_{onset}, °C	T_{max}, °C	T_{end}, °C	Heat of process, J/g	Mass change, %
TIP	0	-	242.3	268.5	296.8	684.89	4.29234
		CH	234.2	262.5	301.3	705.63	4.10035
		nH	235.6	253.5	302.9	140.21	3.07062
		DE	234.9	255.0	287.4	168.64	2,63411
	72	-	233.4	261.6	294.9	644.77	3.87164
		CH	242.5	271.6	301.5	790.12	4.55605
		nH	239.5	254.0	351.0	140.50	3.19938
		DE	228.9	254.0	293.1	451.66	3.31962
TBOT	0	-	246.4	270.3	310.6	754.06	5.29818
		CH	244.3	269.1	312.3	1422.41	7.11736
		nH	244.8	270.1	315.3	964.61	7.03502
		DE	242.2	269.4	314.0	1573.87	7.23537
	72	-	234.0	263.7	297.4	761.72	5.05436
		CH	232.5	259.4	291.7	450.72	2.78393
		nH	229.8	252.7	287.8	259.02	2.90846
		DE	238.9	252.9	351.7	95.11	3.30603
